# Physics Incarnate

**DOI:** 10.1371/journal.pbio.1001022

**Published:** 2011-02-22

**Authors:** Cheryl A. Kerfeld

**Affiliations:** 1 Department of Energy Joint Genome Institute, Walnut Creek, California, United States of America; 2 Department of Plant and Microbial Biology, University of California, Berkeley, California, United States of America

**Figure pbio-1001022-g001:**
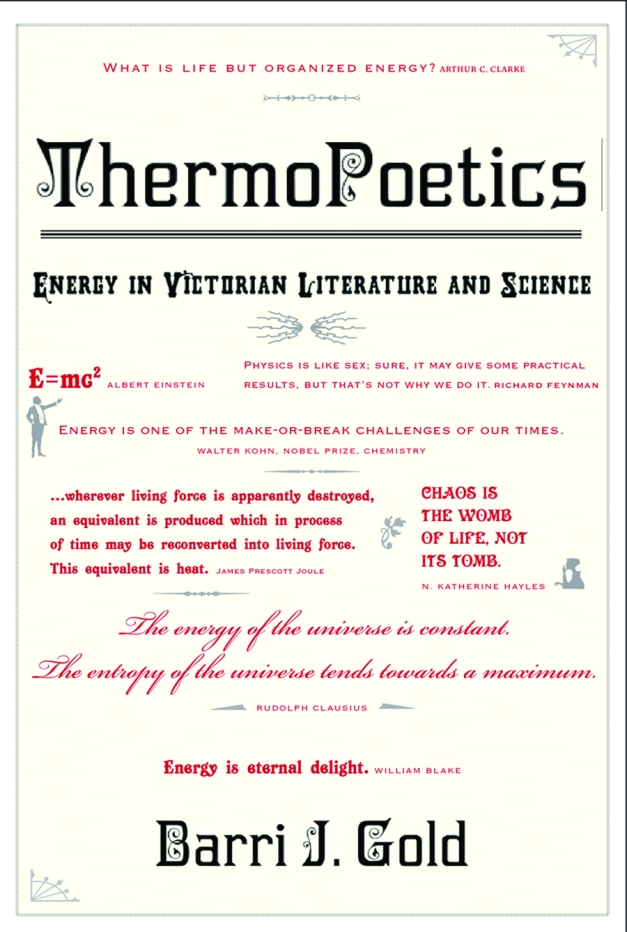
Gold BJ (2010) ThermoPoetics: Energy in Victorian Literature and Science. Cambridge (Massachusetts): The MIT Press. 336 p.

[Fig pbio-1001022-g001]Over 50 years ago, C. P. Snow warned that a growing chasm between scientific and literary intellectuals would leave society ill-prepared to solve its most pressing problems. In his now classic lecture (later published as an essay), “The Two Cultures,” Snow—who as a physicist and novelist had special insight into the problem—described the depth of the divide and appeared to chide the willful ignorance of literary scholars.

“A good many times I have been present at gatherings of people who, by the standards of the traditional culture, are thought highly educated and who have with considerable gusto been expressing their incredulity at the illiteracy of scientists,” he wrote. But when Snow asked the indignant humanists if they could describe the Second Law of Thermodynamics, few could. “Yet I was asking something which is about the scientific equivalent of: ‘Have you read a work of Shakespeare's?’” [1]

Bridging the gap between the sciences and humanities, E. O. Wilson declared, is “the greatest enterprise of the mind” [2]. The endeavor takes many forms, from Literary Darwinism [3] to the latest issue of *Leonardo*, the journal of the International Society for the Arts, Sciences and Technology, and most frequently as disquisitions showing how science and the arts are focused on similar preoccupations (e.g., *Einstein*, *Picasso* [4]). In *ThermoPoetics: Energy in Victorian Literature and Science*, Barri Gold, a Professor of English with a B.S. in physics from the Massachusetts Institute of Technology, adds to the genre by tracing how thermodynamics and evolutionary theory suffused works of literature in the last half of the 19^th^ century.

Gold mainly focuses on major works of the Victorian age, her specialty, establishing her thesis with Alfred, Lord Tennyson's *In Memoriam*. “*In Memoriam* has not been properly appreciated as the brilliant work of thermodynamics that it almost certainly is. As such, it allows us to study the literary production of ideas we think of as scientific. *In Memoriam* suggests, if nothing so simple as the influence of poetry on science, at least something more conversational—something I have come to call *ThermoPoetics*, a term intended to suggest mutual influence, common concerns, and even simultaneous discovery.” In Tennyson's working out of his grief over the loss of his Cambridge friend, Arthur Hallam, Gold finds a parallel to the acceptance in the collective cultural imagination of the “shift from first- to second-law dominance.” The first law had grown out of Romanticism of the early 19^th^ century (there is something soothing, god-like, about a force that can never be created or destroyed), whereas the second law provides a metaphor for a loss of power. Embracing the second law allows Tennyson to transform the energy of his fervent friendship into a more ubiquitous but less potent form. According to Gold, “God comes to look remarkably like a heat sink,” in which Tennyson can diffuse the homoerotic attraction that literary scholars have readily found troubling him in the poem. What Gold calls the “consolation of thermodynamics,” finding transformation where the senses perceive loss and waste, also makes the poem a “progressivist evolutionary narrative.” Prerequisite to accepting this claim is the assumption that something was in the air that crystallized into Charles Darwin's *The Origin of Species*, because *In Memoriam* predates publication of *The Origin of Species* by ten years. Indeed, *ThermoPoetics*, although it doesn't advertise it in the title, is almost equally concerned with the influence of evolutionary theory as thermodynamics on Victorian literature. For example, in *In Memoriam*, Gold sees these “two scientific subtexts…entangled….But evolutionary biology, the poem suggests, is able to transform waste into progress because it grows up alongside of and in conversation with the notion of transformation central to the development of thermodynamics.”

Gold next takes up Charles Dickens's *A Tale of Two Cities* and *Bleak House*. The latter is a novel “almost overwhelmed by entropy.” Again Gold is forced to ask, and answer, “How, we may reasonably ask, is this possible? After all, the term *entropy* was a decade away.” She traces Dickens's thermodynamic sensibilities in the novels' imagery and themes and draws parallels to the writing of Victorian philosopher Herbert Spencer (who coined the phrase “survival of the fittest”). Gold focuses on how most of the numerous characters in *Bleak House* are wasting time and energy, only to be countered by the actions of the heroine, its housekeeper, Esther Summerson. “We are introduced to her in a chapter called ‘A Progress,’ which suggests how much her ability to do useful work bucks the larger entropic trends of the novel. Her name, evoking both a star and a season, suggests her close association with light and heat….[W]hat draws so many to her is her warmth.” Esther is a “domestic engine” and foreshadows what Gold identifies as an “entropic individual,” “one who is particularly skilled at concentrating and using energy.”

The dark side of the entropic individual is explored in the final chapter of *ThermoPoetics*, “Bodies in Heat.” Many late Victorian literary characters—Dr. Jekyll, Dorian Gray, Dracula—live within a rather familiar state of affairs: “A self-consciously entropic individual, Dr. Jekyll understands the creation of local order—his upright moral successful self—to be dependent on the increase of disorder elsewhere. We may understand Hyde as the increase in entropy necessary to the higher (energy) state enjoyed by the upright, vice-reduced Dr. Jekyll.” Perhaps hypocrisy has a deep thermodynamic explanation.

In Bram Stoker's *Dracula* (published in 1895) Gold finds the full range of Victorian obsessions that come with conquering the world (British imperial expansion), mastering nature (the industrial revolution), and the rise of the New Woman as instantiations of thermodynamic and evolutionary preoccupations. Dracula is “a ‘coming race’ unto himself, he represents at once the colonized and the colonizer.” He is apparently exempt from the second law, and his “vampiric technology” is a model of efficiency: “Dracula accomplishes a remarkable amount of work. He is, after all, producing vampires at an alarming pace—through a process with implications at once imperial, reproductive, sexual, and energetic….By contrast, the vampire hunters seem wasteful, enervated, and extremely slow to reproduce.…Their mistake, as Dracula points out, is that they spread these energies too thinly; ‘they should have kept their energies for use closer to home.’” Dracula is, instead, a model of one who can “concentrate diffuse energy into a usable form…ultimately transforming energy into matter itself.” Gold points to Stoker's imagery of the vampires' preferred mode of locomotion: Dracula moves through the novel as steam, congealing before Mina Harker, in bed with her husband, as “a ‘white face bending over me out of the mist’”; when her husband was trapped at Castle Dracula, he gets a glimpse of the resident female vampires: “those awful women growing into reality through the whirling mist in the moonlight.”

The narrative method of *Dracula* is based on the character Mina Harker's ability to accumulate and organize information. This, as Joan Acocella observed in a *New Yorker* review of a recent edition of *Dracula*, is why the novel is a great work of art: the power of *Dracula* is that it deploys “great and volatile forces within a very tight structure” [5]. The narrative method involves Mina assembling “a collage of purportedly authentic documents, most of them in the first person. Many of the materials are identified as excerpts from diaries of the main characters. In addition, there are letters to and from these people—but also from lawyers, carting companies, and Hungarian nuns—plus telegrams, ‘newspaper’ clippings, and a ship's log” [5]. Mina, too, is an entropic individual, her “demonic capacity to consolidate energy thus converges with her capacity to consolidate information.” Gold likens Mina to James Clerk Maxwell's demon—the imaginary construct that “could sort hot particles from cold, thus decreasing the entropy of the system…[T]he demon possesses the capacity for infinitesimally minute observation.”

Gold asks in the introduction, “Are all the striking parallels we will explore…*just* coincidence?” Unlike Tennyson, whose interests in science are well known, Gold doesn't provide evidence of Stoker's familiarity with thermodynamics and evolutionary theory. And, as is often the case in the arguments for mutual influence, when all told, there is much less evidence for the influence of the arts on science. *ThermoPoetics* contains one example of a letter of Maxwell's in which he makes an allusion to *In Memoriam*, but then again, Maxwell may have been an outlier: he also wrote nerdy poetry, some of which is included here. One might also ask, are these parallels really striking? And, more broadly, are there really two cultures? Is this just an academic question? Perhaps it should be, and not just in the faculty center, but in the classroom, where students could test hypotheses like those put forward by *ThermoPoetics*. Literary analysis and scientific research are both about interpreting data. Both use metaphors as descriptive tools. But one essential difference between the sciences and humanities is that in the latter interpretations of data (e.g., structural and literary devices of a novel) are often indifferent to one another—we can have a Freudian or a Feminist or a ThermoPoetic reading of *Dracula*, each can seem valid without requiring refutation of the others. Teaching *ThermoPoetics* and its primary sources, comparing interpretations and discussing their relative merit in the context of the science alluded to provides a chance for life sciences students to interpret data and on a different scale. Is the scientific reading as valid as any other? Because one cannot be entirely sure, this sort of liminal territory is an ideal place to exercise critical thinking skills. And it offers an opportunity to explore how metaphor is used in science as well as in literature. Awareness of the use of metaphor in biology, like protein “folding” [6] or “bacterial charity” [7], can help develop a sensitivity to how the language of macroscopic description can lead to new hypotheses but can also limit or mislead [6],[8]. Likewise, books like *ThermoPoetics* could be used in courses for non-science majors, translating complex scientific concepts presented in typical textbooks into qualitative descriptions. Exploring hypothetical literary embodiments of the principles of science provides an opportunity to convey them. Gold points to this potential, in this way science and literature can “collaborat[e] in the shaping and circulation of scientific fact.”

The potential for such a collaboration has developed since the gatherings Snow attended in the 1950s. Gold describes the relationships between science and literature “part of a larger conversation, working out of similar concerns”; both grow from the drive to organize, to make sense of experience. While biology is the study of the various ways organisms have evolved to withstand the unifying effects of entropy, literary works organize and explain experience on a different scale; what both offer are hypotheses. The most enterprising use of books like *ThermoPoetics* is in circulating the scientific view of the world and raising awareness in the next generation of scientists of the role of language and metaphor in shaping scientific interpretations. Perhaps this is another way in which the arts can influence science.
